# Lessons from the development process of the Afghanistan integrated package of essential health services

**DOI:** 10.1136/bmjgh-2023-012508

**Published:** 2023-09-28

**Authors:** Sayed Ataullah Saeedzai, Karl Blanchet, Ala Alwan, Najibullah Safi, Ahmad Salehi, Neha S Singh, Gerard Joseph Abou Jaoude, Shafiq Mirzazada, Wahid Majrooh, Ahmad Jan Naeem, Jolene Skordis-Worral, Zulfiqar A Bhutta, Hassan Haghparast-Bidgoli, Fahrad Farewar, Isabelle Lange, William Newbrander, Ritsuko Kakuma, Teri Reynolds, Ferozuddin Feroz

**Affiliations:** 1 M&E HIS, Ministry of Public Health, Kabul, Afghanistan; 2 Global Health Development, University of Geneva Faculty of Medicine, Geneva, Switzerland; 3 Faculty of Public Health and Policy, London School of Hygiene & Tropical Medicine, London, UK; 4 Health System Development, WHO Country office for Afghanistan, Kabul, Afghanistan; 5 Institute for Global Health, University College London, London, UK; 6 Geneva Centre of Humanitarian Studies, Faculty of Medicine, University of Geneva, Geneve, Switzerland; 7 Ex- Ministry of Public Health, Kabul, Afghanistan; 8 Ministry of Public Health, Kabul, Afghanistan; 9 Institute for Global Health, UCL, London, London, UK; 10 Centre for Global Child Health, The Hospital for Sick Children, Toronto, Ontario, Canada; 11 Management Sciences for Health, Cambridge, Massachusetts, USA; 12 Centre for Global Mental Health, London School of Hygiene & Tropical Medicine, London, UK; 13 Integrated Health Services, World Health Organization, Geneva, Switzerland

**Keywords:** Public Health, Health policies and all other topics, Health economics, Health services research, Health policy

## Abstract

In 2017, in the middle of the armed conflict with the Taliban, the Ministry of Public Health decided that the Afghan health system needed a well-defined priority package of health services taking into account the increasing burden of non-communicable diseases and injuries and benefiting from the latest evidence published by DCP3. This leads to a 2-year process involving data analysis, modelling and national consultations, which produce this Integrated Package of Essential health Services (IPEHS). The IPEHS was finalised just before the takeover by the Taliban and could not be implemented. The Afghanistan experience has highlighted the need to address not only the content of a more comprehensive benefit package, but also its implementation and financing. The IPEHS could be used as a basis to help professionals and the new authorities to define their priorities.

**Summary box**

- The development of a priority package in a country requires evidence and political negotiation.
- In Afghanistan, the leadership from the Ministry of Public Health helped build trust, ownership and consensus amongst national actors.
- Afghanistan requires to introduce basic management of diabetes and hypertension and emergency care to better address the current burden of disease.

## Introduction

 Despite an increasing number of armed conflict attacks on civilians since 2015, Afghanistan is on the path to universal health coverage (UHC).^1^ Between September 2017 and August 2021 (prior to the arrival of the Taliban in power), the Ministry of Public Health (MoPH) set up context-specific health, disease and inter-sectoral priorities. This work was carried out within the framework of Afghanistan’s National Health Policy 2015–2020,^2^ which includes revising its basic package of health services (BPHS) and essential package of health services (EPHS) using data from a number of national surveys, reports, journal articles, a costing study and the strengthening of coordination and cooperation with key partners and line ministries. This work was finalised prior to the arrival of the Taliban regime in August 2021 and was not implemented by the Taliban regime.

The context for the development of a revised health package is one in which the Afghan government, since 2002, has achieved substantial improvements in the health status of its population despite serious episodes of insecurity. Between 2000 and 2017, the maternal mortality ratio reduced from 1100 to 638 deaths per 100 000 live births,^2^ and under-five mortality has reduced from 257 to 55 per 1000 live births between 2000 and 2018.^3^

There is clear evidence that the high level of insecurity in some provinces during the pre-Taliban regime period had a negative effect on the delivery and coverage of health services, especially for maternal health and childhood vaccines,^4^ which was later further exacerbated by sanctions post takeover by the Taliban government. Although all provinces in the country increased the coverage of maternal and child health services between 2005 and August 2021,^5^,^7^ there remained significant differences between the poorest and the wealthiest populations, between rural and urban areas, and between provinces in terms of health outcomes and utilisation and coverage of health services.^8 9^ Direct out-of-pocket expenditure by households was also high nationally, accounting for 76.5% of total health expenditure in 2018. Donors and the government contributed to 19.7% and 3.9% of total health expenditure in 2018, respectively.^10^

Key weaknesses in population health observed in Afghanistan since 1990 were the high burden of communicable diseases, poor status or maternal and newborn health, nutritional conditions and largely neglected non-communicable diseases (NCDs).^11^ Among NCDs, ischaemic heart disease, congenital defects and cerebrovascular disease all ranked among the leading causes of premature death,^12^ with the additional high burden of mental health disorders.^13 14^

In 2014, injuries from conflict and road injuries ranked second and fifth, respectively, as causes of premature death.^11^ Furthermore, deaths from conflict and terror notably rose by almost 1200% between 2005 and 2016.^12^ 2017 recorded the highest number of civilian casualties from suicide and complex attacks in a single year in Afghanistan since the United Nations mission in the country began systematic documentation of civilian casualties in 2009. Suicide and complex attacks accounted for 22% of all attacks with 16% of the casualties taking place in Kabul in 2017. In just one attack in the city on 31 May 2017, over 200 people were killed and nearly 600 injured.^15^

### Priority health packages in Afghanistan

In 2001, after the end of the first Taliban regime, the MoPH had the challenging task of rebuilding the health system including how best to address the key health challenges in the country; especially given that its population’s maternal mortality and child mortality rates represented the highest mortality rates in the world.^16^ In 2002/2003, the MoPH designed a unique package of health services that helped bring coherence among the health stakeholders in what was then a fragmented health system. Towards the end of 2003, the MoPH supported by its international partners, put in place the BPHS for the primary healthcare level throughout the country. This was followed in 2005 by the Essential Package of Health Services (EPHS) for hospitals up to provincial level.^17^

The MoPH and health economists included in the Expert Committee advising the MoPH estimated that US$235M were spent by government and donors on the BPHS and EPHS in 2018, equivalent to US$6.7 per capita. The BPHS accounted for 72% (US$172M) of total spending, whereas the EPHS accounted for around 28% (US$63M) of total spending.^18^ Maternal and child health accounted for around 45% of total BPHS spending. Combined, government and donor spending on the BPHS and EPHS averted an estimated 1.04M disability-adjusted life years (DALYs). Almost 60% (605 000) of DALYs averted by the BPHS and EPHS were related to maternal and child health interventions.^19^

**Figure 1:**
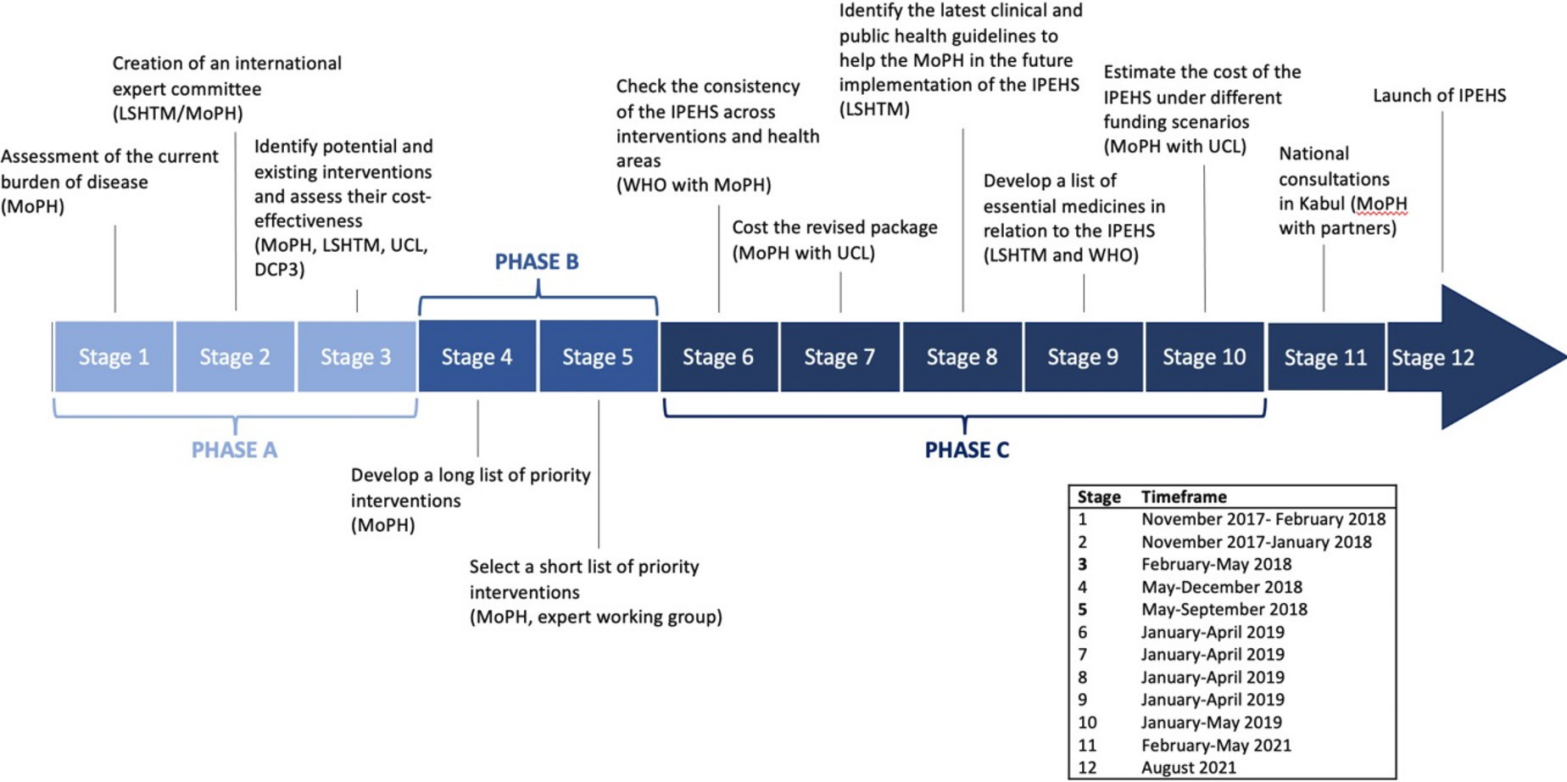
The timeline of the development process of the IPEHS in Afghanistan. DCP3, Disease Control Priorities 3; IPEHS, Integrated Package of Essential Health Services; LSHTM, London School of Hygiene and Tropical Medicine; MoPH, Ministry of Public Health; UCL, University College London, universal health coverage.

In 2018, the MoPH decided that the BPHS and EPHS needed revising in light of the increase burden of disease since 2006 related to NCDs (+2.5% annually) and injuries (+4.4% annually), the international drive towards UHC.^20^ and the publication of DCP3.^21^ In August 2021 (see figure 1), the new priority package, the Integrated Package of Essential Health Services (IPEHS) was finalised.

## Priority setting processes

### The various trade-offs

The difficult decisions made in Afghanistan when starting working on the IPEHS in 2018 were about responding both to the epidemiological transition and level of violence generated by armed conflict, while maintaining gains in maternal and child health, ensuring equitable access to interventions and providing financial protection—within a highly constrained government and donor budget envelope. Two key questions for the MoPH guided the priority setting process. First, in the current BPHS and EPHS, which interventions are no longer justified as a top priority and which additional health interventions are needed? Second, how to ensure the new package of health services is accessible to the most underprivileged that is, the poorest and the groups of populations living the furthest from primary healthcare facilities?

Priority setting in Afghanistan between 2028 and 2021 was about making trade-offs not only between different health interventions from different disease groups but also between health services, public health interventions and interventions tackling determinants of health. These decisions carried with them value judgements and efficiency (cost-effectiveness)-equity trade-offs. A priority setting process usually takes place in an environment where societal values are at stake and where tensions exist between different perspectives and interests.^22^ This process required legitimacy in order to gain any prospect of public and political acceptance. As a result, all decision was justified with rigorous documentation to make sure that every step in the process was cumulative from the previous one.^23^

In terms of governance, the MoPH, led by the Minister of Public Health, drove the revision process. In their role of overseeing this activity, the MoPH core team created and managed nine in-country Working Groups and ‘integrated expert opinion from members of the Ministry and the local stakeholder community including international organisations such as United Nations agencies. In Afghanistan, nine multistakeholder Working Groups were set up according to health domains (reproductive, maternal, child and adolescent health; mental health; surgery; cardiovascular health; infectious disease; surgery; cancer; palliative care; rehabilitation and inter-sectoral policy) to provide expertise in reviewing the shortfalls in the BPHS and EPHS. An advisory mechanism in the form of an international Expert Committee was put in place to maximise the use of data and evidence, ensure the adequacy of the methodology, encourage creativity in data analysis and provide accountability for use of the results by the Afghan government as well as by national and global stakeholders’.[24 Page 3]

### A multi-criteria approach

MoPH adopted a multicriteria approach to enable them to have a fair, transparent and mutual process to set priorities.^24^ This approach was based on the following principles: (1) use of the latest global and national evidence on burden of disease and cost-effectiveness of interventions, (2) well-defined selection criteria agreed by all key stakeholders, (3) transparent and documented process of selecting interventions and (4) recognition that decisions made are reasonable, combining both analysis of evidence and expert discussions.

The selection criteria defined by the Expert Committee in May 2018 to guide decisions of MoPH and experts included the following: (1) effectiveness: What has been proven to work? (2) local feasibility: local resources exist to deliver? Are there staff in place? Are they trained? Is the intervention supported by existing infrastructure? (3) affordability: Are new drugs and equipment required? Is there a large setup cost?; and (iv) Equity: Will the intervention improve access to care? For whom?

The Expert Committee and MoPH also agreed on a set of priority conditions and risk factors to address the current burden of disease in Afghanistan. The priority conditions included reproductive, maternal, newborn and child health, injuries (conflict and road traffic accidents), mental health (substance use, suicide, posttraumatic stress disorder), cardiovascular diseases (heart attack, stroke), undifferentiated emergency presentation (difficulty breathing, shock, meningitis, diarrheal disease, lower respiratory diseases) and diabetes. The priority risk factors identified included undernutrition, over-nutrition, smoking, water sanitation and hygiene, air pollution and hypertension.

The MoPH designed a flexible process to examine in-depth the bigger picture that is internal and external to the setting of priorities by the institution to reflect the connection and relationship between the different parts of the health system, and in doing so:

1. MoPH research teams conducted an analysis of the health needs and the health system capacity.
2. An expert committee was established, chaired by the Minister of Public Health and composed of 12 national and international experts including from the DCP3 task force.
3. Nine local working groups were formed (one for each of the nine health volumes of DCP3),^21^ to create an initial draft of priority interventions based on field experience.
4. A number of opportunities created for a wide range of stakeholders to help decide the priorities through consultative workshops and meetings with NGOs, UN agencies, Donors and Presidential office.
5. Defined clear selection criteria for the setting of priority interventions and opportunities.
6. Costed the existing and new package of health services and the identification of relevant global cost-effective interventions.
7. Projections of the fiscal space between 2018 and 2030 conducted on different scenarios.
8. Enhancing advocacy and negotiation to mobilise domestic revenue.
9. Rigorously examined the short-term and long-term implications of the new package of health services and developing relevant implementation approaches and systems including a tailored monitoring and information system.

At the same time, MoPH determined which of the DCP3 early intersectoral policy interventions was addressed as a priority using standardised and transparent criteria. It also worked on minimising financial risks to people, especially the poor in Afghanistan.

The priority setting process was conducted within the available and projected fiscal space. According to the Ministry of Finance and the 2020 National Health Accounts, more than half (52%) of the national budget was funded by foreign aid, 44.8% by domestic revenue and 3.2% by loan.^25^ From the total budget, 5% was allocated to MoPH, of which about 79% was funded by donors covering the BPHS and EPHS. Through the MoPH’s budgetary prospect exercise, three possible realistic scenarios for budget expansion were developed in order to cover the potential expansion of services provided under the High Priority Programme for Afghanistan.^26^ Based on stable support from international donors, stable economic growth and a slight reduction in out of pocket expenditure, it was estimated that in a low variant projection, the per capita expenditure will increase by one per cent per year. In a medium variant projection, it was estimated that the total health spending per capita will increase by 5%, and in a high variant projection by 8%.^26^ Of course, these projections did not include the scenario that the Taliban would take over in August 2021.

## Analysis and tool

### The use of DCP3 data

The third edition of *Disease Control Priorities published between 2015 and 2018 in nine volumes* provides a review of evidence on cost-effective interventions to address the burden of disease in low and middle-income countries.^21^ It does so by drawing on systematic reviews of economic evaluations, epidemiological data and clinical effectiveness studies, and on the expertise and time of over 500 authors.^27^ While DCP3 data are generally considered thorough and to have been constituted in a transparent manner, considerable adaptation must be undertaken when applying it at the country level, especially in those countries, like Afghanistan, where contextually adapted evidence was especially needed given the complexity brought about by sectarian violence and armed conflict. National health officials are advised by DCP3 that its packages of interventions needed to be modified based on local priorities, and that country-specific analyses as to costs and impact should be carried out.

To inform each health system building block, team members consulted additional sources, including the most recently available national health information systems data and results from the^28^ Mental Health survey and other national surveys.^29^ To develop the list of interventions, working groups compared the DCP3 list of interventions with the existing BPHS and the EPHS. The MoPH decided that the revised package of health services would be unique from community level to provincial level—instead of two distinct packages. This involved prioritising the interventions in DCP3 and assigning them to the different categories of health system level, categorised by health facility type. Contextual knowledge and specialist assessment as to which interventions would be possible given government and partner support at each level were critical for this task.

### DALY-driven rationale

DALYs are a measure of the burden of disease accounting for the number of years lost due to ill health, disability or early death. DALYs ‘measure the gap between a population’s health and a hypothetical ideal for health achievement’^30^ and are used in setting health research priorities, identifying disadvantaged groups and targeting health interventions. While estimates, projections and modelling that are based on mortality—how many deaths could be averted due to a health service being offered—are popular and compelling, unlike DALYs they do not capture morbidities such as chronic diseases, mental health, injuries and disabilities, that will have an impact on quality of life.

The Expert Committee took the decision to use DALYs through the Health Interventions Prioritisation tool (HIPtool),^31^ a health resource optimisation tool, using context-specific data on burden of disease and intervention cost-effectiveness to help stakeholders identify funding priorities and targets. The reference point of this expert committee consultation, the Essential Universal Health Coverage package published by DCP3, is based on evidence of cost-effectiveness, presenting data in the form of ‘cost per DALY averted’ (an incremental cost-effectiveness ratio, ICER).^21^ DALYs provided a single measure for which to compare interventions across the entire BPHS and EPHS packages. Given the amount of diseases and interventions considered, it is important to note that results might have been less clear to interpret if a variety of outputs were used.

### Summary of analysis findings

In the first comprehensive list, 149 interventions were included for consideration. For the international expert committee meetings, HIPtool generated estimates of DALYs averted by: (1) existing spending, (2) additional spending projections based on fiscal space assessments, (3) scaling-up existing Reproductive, Maternal, Newborn and Child Health (RMNCH) interventions in the package and (4) optimised spending based on intervention cost-effectiveness and burden of disease. The HIPtool optimised spending scenario supported recommendations on the inclusion of emergency and trauma care as well as cost-effective mental health interventions in the IPEHS package.

The IPEHS was organised by seven platforms of the health system: (1) community health post; (2) mobile health teams (MHT); (3) subhealth centre (SHC); (4) basic health centre (BHC); (5) comprehensive health centre (CHC); (6) first referral hospital and (7) second referral hospital. In order to highlight the level of integration and continuum between the various levels of the health system, the interventions were defined by level based on the resources and skills available at the level with an explicit link with the previous or next level of referral (see Annex 1 for the full list of IPEHS interventions).

Nine domains were defined to help structure the interventions: (1) reproductive, maternal and newborn health; (2) child and adolescent health and development; (3) infectious diseases; (4) chronic NCDs; (v) mental, neurological and substance use disorders; (vi) emergency care; (vii) surgical interventions; (viii) palliative care and (ix) rehabilitation.

These nine domains were completed by 11 population-based interventions such as mass media campaign promoting healthy diet and physical exercise or preparedness strategy in case of infectious disease outbreak.

Finally, the IPEHS was composed of 15inter-sectoral interventions such as regulate transport, industrial, power and household generation emissions to reduce air pollution or ban smoking in public places.

### Cost of IPEHS

Healthcare access, quality and outcomes vary widely across geographies in Afghanistan. Variations in the financing and provision of healthcare services along with population displacements, geographic remoteness, difficult terrain, sociocultural isolation and health awareness contribute to these differences. To address this, a number of provinces were carefully selected for inclusion in the cost analysis to achieve good geographic spread and sufficient representation from each region: Dikundi, Faryab, Takhar, Nangarhar, Paktya, Urzgan and Herat based on geographical representations from Central, North West, North East, East, South, South West, and West, respectively.

The BPHS cost analysis was carried out using the Cost Revenue Analysis Tool Plus (CORE Plus) for MHT, SHC, BHC, CHC and district hospital (DH) levels of the health system. Expenditure data were collected from NGOs from 534 health facilities in seven selected provinces in AFN currency, and it was converted to USD based on an exchange rate of 2020 at 78 AFN.^18^ The studied health facilities covered 21% of the total population in 2020. Provincial hospitals (PH) and higher levels of the health system, for the EPHS, were costed separately using hospital data.

The difference between the costs of BPHS and EPHS and IPEHS 2021 was also assessed to understand the costs of supplementary interventions under IPEHS 2021. The health facilities were categorised into two groups—primary healthcare services and secondary healthcare services, which included PH. The total additional cost of the supplementary interventions was estimated at US$39 141 581. The additional costs of IPEHS compared with BPHS at the primary healthcare level (Community level, Mobile Health Tesm, sub-Health Centre, BHC, CHC, DH) and compared with EPHS at secondary healthcare level (PH and above) were US$30 334 630 and US$8 808 951, respectively. In other words, primary healthcare accounted for 77.5% of the total required increase in IPEHS cost, whereas the cost of the additional secondary service share was 22.5% of the total cost. The overall average per capita cost of IPEHS was US$6.9.^18^

## Methodological limitations

Getting access to data was a tremendous challenge for the working groups and the international expert committee. As a result, consensus panels were applied to capture expert opinion. This approach can synthesise expert opinion when other data are not available. However, such method is prone to various types of biases. Therefore, more studies on benefit-incidence analysis and cost-effectiveness were necessary for future exercises in Afghanistan to better assess implications on equity and allocative efficiency.

Given the number of interventions, project budget and time constraints to meet a policy reform window, no cost-effectiveness study was conducted in Afghanistan for this prioritisation exercise. HIPtool drew on national cost-analysis data, available by intervention and cost-effectiveness data published by DCP3 to estimate existing and potential population health impact for each intervention and for different health packages as a whole. One justification was that DCP3 volumes had just been released providing up-to-date reviews on effectiveness and cost-effectiveness of health interventions at global level—with a focus on low and lower middle income countries. The analysis of these reviews was discussed in the international expert committee to verify the relevance of the DCP3 findings. Using existing evidence and HIPtool enabled us to carry out analyses to quantify trade-offs of different decisions, in terms of population health, iteratively throughout the process and to inform three key discussions on IPEHS design.

The prioritisation exercise was a heavy process mobilising a lot of resources in country and outside. It required more than 2 years to finalise the high-priority package and make sure that concerned parties (senior staff at MoPH, provincial authorities, development partners) were properly engaged. One possibility of reducing these transaction costs could be to regularly update the priority package and organise a review of the package around every 3 years or in line with 5-year national plans.

This prioritisation process greatly benefited from the experience of the two successive ministers as Afghanistan had conducted a similar exercise in 2012. With the arrival of the Taliban, many individuals with high level expertise in Afghanistan left the country. The revision or conduct of such processes in the near future will require political willingness and rebuilding expertise in the country on health economics and public health as well as availability and modality of resource allocation.

## Lessons learnt

The prioritised package, IPEHS, contained 144 health interventions and 14 intersectoral interventions that address the burden of communicable diseases, reproductive, maternal, newborn and child health, chronic diseases and injuries due to armed conflict. It included for the first time cost-effective services for chronic conditions, such as diabetes and hypertension, emergency trauma care and palliative care, while maintaining focus on addressing the high maternal and neonatal mortality rates. The package was finalised in August 2021, just before the Taliban took over the country.

The IPEHS development was supported by Bill and Melind Gates Foundation as well as UN agencies and Sehatmandi donors (World Bank, USAID, European Union, Canada). While there was high-level commitment at the MOPH, the budgetary prospect was very limited and it was met with hesitancy from international donors. The emergence of a new package raised questions among donors on the financial capacity of the government to increase financial commitment to cover the new interventions and ensure no increased out-of-pocket payments.

A set of challenges and needs were identified in revising the health benefits package in Afghanistan. The team faced difficulties in knowing how and when to start the process of revising the BPHS, citing lack of clear vision from the start of what the government thought was most needed in Afghanistan. There was also a clash between the political and health agendas, which led to increased pressure to deliver the revised package before the 2019 elections. This relative short timeline (18 months) to deliver a full revised package leads to a shortened consultation process in country expressed by national stakeholders as a missed opportunity to create ownership. While several governmental departments and provincial health directors were involved in the process of revising the benefit package, there was a realisation that information on the prioritised package was not cascading effectively from top leadership across the health system. Two national consultations were organised in February and May 2021 to overcome this communication gap and receive feedback on the revised package. As a result, the 2019 IPEHS was left aside after the departure of the Minister. It was not until the end of the 2020 that there was revived interest in the IPEHS by the President of Afghanistan. The MoPH decided to finalise the IPEHS by emphasising the national consultation process. University of Geneva was called back to provide guidance and help integrate feedback from national stakeholders into the IPEHS, which resulted in the 2021 IPEHS. A detailed account and review of the priority setting process as a whole was published by Lange *et al.*^23^

Change of MoPH leadership in the middle of the project in 2019 impeded the finalisation of costing the package, its implementation and sustainability. Inadequate commitment and engagement of the Ministry of Finance, low budget allocation and overdependency on donor funding remain major challenges for UHC in Afghanistan. In 2021, the costing of the IPEHS was finalised, but this time, the arrival of the Taliban prevented the MoPH and University of Geneva from developing a realistic implementation plan.

Since the Taliban took control over Afghanistan, implementation of the IPEHS is on hold due to the current political situation. The experience in revising the Afghanistan IPEHS highlighted the need to address not only the development of a more comprehensive benefit package but also its implementation, with careful deliberation on the pre-requisites for implementing and financing the HBP and health systems strengthening. The IPEHS can be used as a foundation to define a new priority package under the Taliban rule.

## Supplementary material

10.1136/bmjgh-2023-012508 online supplemental file 1

## Data Availability

Data are available upon reasonable request.
