# Supplementary material for: Lessons from the development process of the Afghanistan integrated package of essential health services
Source: BMJ Glob Health. 2023 Sep 28;8(9):e012508. doi: 10.1136/bmjgh-2023-012508 (PMC10546159; doi:10.1136/bmjgh-2023-012508)
Supplement: online supplemental file 1 [file bmjgh-2023-012508supp001.pdf]

Islamic Republic of Afghanistan Ministry of Public Health

# Integrated Package of Essential Health Services

**2021**

Health for All

**Ministry of Public Health, Afghanistan**

**Integrated Package of Essential Health Services 2021:**

## Health, Medical, and Surgical Interventions

**Table 1**

| Community health post 16,510* | Mobile health teams 309* | Sub-health centre (SHC) 1,001* | Basic health centre (BHC) 874* | Comprehensive health centre (CHC) 433* | First referral hospital 85* | Second referral hospital 27* |
|---|---|---|---|---|---|---|
| 2 Community health workers (CHWs), one female and one male | *Staff: 1 male physician; 1 Community Midwife; 1 vaccinator; 1 Nutrition Nurse; 1 Driver (with vehicle)<br><br>*Can provide Family Health Home (FHH) as alternative for MHTs in remote areas with difficulties in physical access | *Staff: 1 male nurse; 1 community midwife; 1 cleaner/guard, 1 Nutrition counsellor, 1-2 vaccinators | *Staff: 1 female physician; (To be optional) 1 male nurse; 1 community midwife; 1 pharmacy technician; 1 CHS; 1 Nutrition Counsellor; 2 Vaccinator; 2 cleaner/guard | *Staff: 1 male physician; 1 female physician; 1 male nurse; 1 female nurse; 2 (M, F) psychosocial counsellors (nurse); 2 community midwives; 1 Nutrition counsellor; 1 community health supervisor; 2 vaccinators; 1 laboratory technician; 1 microscopies for TB; 1 pharmacy technician; 1 administrator; 4 cleaners/guards; 2 | *Staff: 1 Hospital Director; 1 Nursing Director; 1 Administrator; 2 Surgeons; 1 Obstetric and Gynaecologist; 1 Pedestrian; 3 General Practitioners; 3 Operating theatre and sterilization (nurse); 5 Midwives; 8 Ward Nurse; 2 Aesthetic Nurse; 2 nurses for emergency room and outpatient department; 2 Physiotherapist; 2 Pharmacist; 2 x-ray technician; 3 Laboratory Technician; 1 blood bank | *Staff: 1 Hospital Director; 1 Nursing Director; 1 Administrator; 4 surgeons; 2 anaesthetist; 4 obstetrician /gynaecologists; 2 paediatricians; 3 medical specialists; 1 ophthalmologist; 1 orthopaedist/traumatologist; 10 general practitioners); 1 dentist; 5 nurses; 9 midwives; 16 ward nurses; 3 anaesthetic nurses; 6 nurses for emergency room and outpatient department; 1 psychiatrist; 2 psychologist |

| | | | | | | |
|---|---|---|---|---|---|---|
| | | | | drivers (with ambulance) | technician, 1 Dental Technician;2 Nutrition counsellor; 2 psychosocial counsellor; 4 Vaccinator; 2 Administration (procurement, accounting, human resource, medical record, clerk); 1Community Health Supervisor; 1 Maintenance; 5 Cleaner, waste management and grounds (gardener); 3 Laundry; 2 Cook; 2 Driver; 4 Guard (porter) | (1 male and 1 female), 2 psychosocial counsellor; 4 physiotherapist; 4 pharmacists; 1 radiologist; 2 x-ray technicians; 4 laboratory technicians; 2 blood bank technician; 2 dental technician; 2 Nutrition counsellor; 4 vaccinators; 2 technical assistants; 4 Administration; 2 Storekeeper; 4 Maintenance; 20 Cleaners, waste management, and grounds (gardeners); 4 Laundry; 4 Cook; 2 tailor, 1 mullah, 4 Drivers (and porters); 2 tailors; 8 Guards |
| *A. Reproductive, Maternal and Newborn Health Interventions = 33 of which 20 are MoPH high priority for implementation* | | | | | | |
| **C1. Family health action groups especially for support when there is domestic violence, for newborn care, and nutrition education | *See also mental health section* | *See also mental health section* | *See also mental health section* | *See also mental health section* | | |

| | | | | | | |
|---|---|---|---|---|---|---|
| ^C2. Provision of appropriate vitamin and mineral supplementation (including vitamin D), iron folic acid tablets, albendazole, and vitamin A to pregnant and lactating women and refer eligible women for tetanus vaccination | MHT1. Provision of mineral supplementation (including vitamin D and calcium), iron folic acid tablets, albendazole, and vitamin A and tetanus vaccination | S1. At least 4 antenatal care visits by pregnant women that includes essential education on maternal health and family planning, support for those experiencing domestic violence, recognition of danger signs for hypertensive disorders and gestational diabetes, promotion of healthy diet and relevant vitamin and mineral supplementation (including vitamin D), iron folic acid tablets, albendazole, and vitamin A. HIV education and counselling, and tetanus vaccination | B1. At least 4 antenatal care visits by pregnant women that includes essential education on maternal health and family planning, support for those experiencing domestic violence, recognition of danger signs for hypertensive disorders and gestational diabetes, promotion of healthy diet and relevant vitamin and mineral supplementation (including vitamin D), iron folic acid tablets, albendazole, and vitamin A. HIV education and counselling, and tetanus vaccination | ^CHC1. Comprehensive antenatal care for complicated pregnancy including management of hypertensive disorders, gestational diabetes, PMTCT of HIV, vitamin and mineral supplementation (including vitamin D), iron folic acid tablets, albendazole, and vitamin A and nutrition interventions | ^DH1. Comprehensive antenatal care for complicated pregnancy, including management of hypertensive disorders, gestational diabetes, PMTCT of HIV, vitamin and mineral supplementation (including vitamin D), iron folic acid tablets, albendazole, and vitamin A and relevant nutrition interventions | ^PH1. Comprehensive antenatal care for complicated pregnancy, including management of hypertensive disorders, gestational diabetes, PMTCT of HIV, vitamin and mineral supplementation (including vitamin D), iron folic acid tablets, albendazole, and vitamin A and nutrition interventions |
| C3. Information on recognition of signs of pre-term labour | ^MHT2. Early detection of pre-term labour and premature rupture of membranes with timely referral and first dose of antibiotic if indicated | ^S2. Early detection of pre-term labour and premature rupture of membranes with timely referral and first dose of antibiotic if indicated | ^B2. Early detection of pre-term labour and premature rupture of membranes with timely referral and first dose of antibiotic if indicated | ^CHC2. Early detection of pre-term labour and premature rupture of membranes with timely referral and first dose of antibiotic if indicated | ^DH2. Management of preterm labour and pre-term pre-labour rupture of membranes with antenatal corticosteroids and antibiotic as indicated | ^PH2. Management of pre-term labour and pre-term pre-labour rupture of membranes with antenatal corticosteroids and antibiotic as indicated |
| | MHT3. Early detection of signs of pre-eclampsia with timely referral | S3. Early detection of signs of pre-eclampsia with timely referral | ^B3. Initial stabilization and management of eclampsia with intra-muscular injection of magnesium sulphate, and transfer to hospital | ^CHC3. Initial stabilization and management of eclampsia with intra-muscular or intravenous loading dose of magnesium | ^DH3. Comprehensive management of eclampsia [FLH4] | ^PH3. Comprehensive management of eclampsia |

| | | | | | | |
|---|---|---|---|---|---|---|
| | | | | sulphate, and transfer to hos[ital | | |
| | ^MHT4. In remote areas, initial treatment of obstetric or delivery complications prior to transfer | ^S4. In remote areas, management of labour and delivery in low-risk women and adolescents (BEmONC), including initial treatment of obstetric or delivery complications prior to transfer | ^B4. Management of labour and delivery in low-risk women and adolescents (BEmONC), including initial treatment of obstetric or delivery complications prior to transfer | ^CHC4. Management of labour and delivery in low-risk women and adolescents (BEmONC), including initial treatment of obstetric or delivery complications prior to transfer | ^DH4. Management of labour and delivery in high-risk women and adolescents including caesarean delivery (CEmONC) | ^PH4. Management of labour and delivery in high-risk women and adolescents including caesarean delivery (CEmONC) |
| ^C4. Promotion of kangaroo care and early breastfeeding and helping babies breathe interventions | MHT5. Helping babies breathe interventions | ^S5. Helping babies breathe interventions | ^B5. Helping babies breathe interventions | ^CHC5. Helping babies breathe interventions and management of newborn complications, including jaundice, neonatal meningitis, and other very serious infections requiring continuous supportive care (intravenous fluids, oxygen, etc.) | ^DH5. Helping babies breathe interventions and management of newborn complications, including jaundice, neonatal meningitis, and other very serious infections requiring continuous supportive care (intravenous fluids, oxygen, etc.) | ^PH5. Helping babies breathe interventions and management of newborn complications, including jaundice, neonatal meningitis, and other very serious infections requiring continuous supportive care (intravenous fluids, oxygen, etc.) |
| C5. Post-natal home visit within 24 hours | ^MHT6. Referral for clinical signs of pre and post natal maternal and neo-natal danger signs especially maternal sepsis | ^S6. Early recognition and referral for clinical signs of pre and post natal maternal and neo-natal danger signs especially maternal sepsis | ^B6. Early recognition and referral for clinical signs of maternal sepsis | ^CHC6. Early recognition and referral for clinical signs of maternal sepsis | ^DH6. Management of maternal sepsis, including early detection | ^PH6. Management of maternal sepsis |
| C6. Post-natal reproductive health visit in home or family health house (FHH) that includes distribution of family planning commodities, resumption of | MHT7. Distribution of family planning commodities | S7. Post-natal visit that includes advice about birth spacing, family planning, resumption of sexual activity and pelvic floor | B7. Post-natal visit that includes advice about birth spacing, family planning, resumption of sexual activity, and pelvic floor exercises, and | CHC7. Post-natal visit that includes advice about birth spacing, family planning, resumption of sexual activity, and pelvic | DH7. Post-natal visit that includes advice about birth spacing, family planning, resumption of sexual activity, and pelvic floor exercises | PH7. Post-natal visit that includes advice about birth spacing, family planning, resumption of sexual activity, and pelvic floor exercises |

| | | | | | | |
|---|---|---|---|---|---|---|
| sexual activity and pelvic floor exercises | | exercises, and complete the TD vaccine schedule | complete the TD vaccine schedule | floor exercises, and complete the TD vaccine schedule | | |
| | MHT8. Counselling and referral for miscarriage or incomplete, or missed abortion | S8. Counselling and referral for miscarriage or incomplete, or missed abortion | ^B8. Management of miscarriage or incomplete or missed abortion and post abortion care [HC2] | ^CHC8. Termination of pregnancy for medical reasons including by manual vacuum aspiration | ^DH8. Surgical termination of pregnancy for medical reasons by manual vacuum aspiration and dilation and curettage | ^PH8. Surgical termination of pregnancy for medical reasons by manual vacuum aspiration and dilation and curettage |
| | | | | | ^DH9. Operative treatment for ectopic pregnancy or ovarian cyst torsion | ^PH9. Operative treatment for ectopic pregnancy or ovarian cyst torsion [ |
| | | | | | ^DH10. Hysterectomy for uterine rupture or intractable postpartum haemorrhage | ^PH10. Hysterectomy for uterine rupture or intractable postpartum haemorrhage |
| C7. Provision of condoms and hormonal contraceptives including emergency contraceptives | MHT9. Administration of long-acting contraceptive methods | ^S9. Referral for, or where available, administration of, long-acting contraceptive methods | ^B9. Insertion and removal of long-acting contraceptives | ^CHC9. Insertion and removal of long-acting contraceptives | ^DH11. Surgical methods of contraception including tubal ligation and vasectomy | ^PH12. Surgical methods of contraception including tubal ligation and vasectomy |
| | | | | | DH12. Repair of obstetric fistula | PH13. Repair of obstetric fistula |
| | | | | ^C10. Post gender-based violence care, including provision of emergency contraception, and rape response referral (medical and judicial) [ | ^DH13. Post gender-based violence care, including provision of emergency contraception, and rape response referral (medical and judicial) | ^PH14. Post gender-based violence care including provision of emergency contraception, and rape response referral (medical and judicial) |
| C8. Promotion of maternal nutrition by nutrition situation assessment through BMI, MUAC, and height, weight measurement | MHT10. Promotion of maternal nutrition by nutrition situation assessment through BMI, MUAC, and height, weight measurement and giving | SHC10. Promotion of maternal nutrition by nutrition situation assessment through BMI, MUAC, and height, weight measurement and giving | B10. Promotion of maternal nutrition by nutrition situation assessment through BMI, MUAC, and height, weight measurement and giving | CHC11. Promotion of maternal nutrition by nutrition situation assessment through BMI, MUAC, and height, weight | DH14. Promotion of maternal nutrition by nutrition situation assessment through BMI, MUAC, and height, weight measurement and giving | PH15. Promotion of maternal nutrition by nutrition situation assessment through BMI, MUAC, and height, weight measurement and giving |

| | | | | | | |
|---|---|---|---|---|---|---|
| and giving proper nutrition counselling accordingly | proper nutrition counselling accordingly | proper nutrition counselling accordingly | proper nutrition counselling accordingly | measurement and giving proper nutrition counselling accordingly | proper nutrition counselling accordingly | proper nutrition counselling accordingly |
| *B. Child and Adolescent Health and Development Interventions = 12 of which 7 are MoPH high priority for implementation* | | | | | | |
| *For treatment of acute infections see infectious disease section and emergency care section* | | | | | | |
| C9. Monthly growth monitoring and health promotion for children under 5 with referral for malnutrition or other complications and screening (oedema, MUAC measurement) | MHT11. Screening (oedema, MUAC, weight per height measurement) and referral for malnutrition or other complications | S11. Screening (oedema, MUAC, weight per height measurement) and monthly growth monitoring and health promotion for children under 5 with referral for complicated malnutrition cases | B11. Screening (oedema, MUAC, weight per height measurement) and monthly growth monitoring and health promotion for children under 5 with referral for complicated malnutrition cases | CHC12. Screening (oedema, MUAC, weight per height measurement) and monthly growth monitoring and health promotion for children under 5 with referral for complicated malnutrition cases | DH15. Screening (oedema, MUAC, weight per height measurement) and monthly growth monitoring and health promotion for children under 5 with referral for malnutrition or other complications | PH16. Monthly growth monitoring and health promotion for children under 5 with referral for malnutrition or other complications |
| C10. Recognition of danger signs per IMCI protocols and referral as indicated | MHT12. Recognition of danger signs per IMCI protocols and referral as indicated | S12. Recognition of danger signs and management per IMCI protocols and referral as indicated | B12. Recognition of danger signs and management per IMCI protocols and referral as indicated | CHC13. Recognition of danger signs and management per IMCI protocols and referral as indicated | DH16. Triage of children on arrival with validated instrument (e.g., WHO/ICRC triage tool) and syndromic management as indicated | PH17. Triage of children on arrival with validated instrument (e.g., WHO/ICRC triage tool) and syndromic management as indicated |
| | | S13. Routine visits to promote early child development, monitoring for expected developmental milestones and referral for delay in development | B13. Improve early child development through introduction of early child development services | CHC14. Improve early child development through introduction of early child development services | DH17. Targeted therapeutic programmes for children referred with developmental delays including motor, sensory, and language stimulation | PH18. Targeted therapeutic programmes for children referred with developmental delays including motor, sensory, and language stimulation |
| C11. Promotion of relevant childhood nutrition interventions | | S14. Promotion of relevant childhood nutrition interventions and | ^B14. Management of moderate and severe acute malnutrition | ^CHC15. Management of moderate and non-complicated severe acute malnutrition | ^DH18. Management of moderate and severe acute malnutrition associated with serious infection | ^PH19. Management of moderate and severe acute malnutrition associated with serious infection |

| | | | | | | |
|---|---|---|---|---|---|---|
| | | management of moderate acute malnutrition | | associated with serious infection | | according to clinical feeding protocols |
| C12. Promotion of initial breastfeeding, complementary breastfeeding, food demonstration, complementary feeding and micronutrient powder distribution and maternal nutrition | MHT13. Promotion of initial breastfeeding, complementary breastfeeding, food demonstration, complementary feeding and micronutrient powder distribution and maternal nutrition | SHC15. Promotion of baby friendly initiative (BFI) | B15. Promotion of baby friendly initiative (BFI) | CHC16. Promotion of baby friendly initiative (BFI) | DH19. Promotion of baby friendly initiative (BFI) | PH20. Promotion of baby friendly initiative (BFI |
| ^C13. Education on hand washing and safe disposal of children's faeces | ^MHT14. Basic treatment of acute diarrhoea including oral fluids and zinc tablet | ^S16. Basic treatment of acute diarrhoea including oral fluids | ^B16. Basic treatment of acute diarrhoea including oral fluids | ^CHC17. Advanced treatment of severe diarrhoea including IV fluids | ^DH20. Advanced treatment of severe diarrhoea including IV fluids | ^PH21. Advanced treatment of severe diarrhoea including IV fluids |
| C14. Periodic outreach initiatives for age appropriate child vaccination or refer for vaccination | ^ MHT15. Routine age appropriate immunization or refer for vaccination | ^S17. Routine age appropriate immunization | ^B17. Routine age appropriate immunization | ^CHC18. Routine age appropriate immunization | ^DH20. Routine age appropriate immunization | ^PH22. Routine age appropriate immunization |
| C15. Promotion of child safety including prevention of road traffic injury, falls and poisoning | | S18. Promotion of child safety including prevention of road traffic injury, falls and poisoning | B18. Promotion of child safety including prevention of road traffic injury, falls and poisoning | CHC19. Promotion of child safety including prevention of road traffic injury, falls and poisoning | | |
| | | | B19. Early identification of lead poisoning and counselling of families to reduce or prevent exposure to lead in the environment | CHC20. Early identification of lead poisoning and counselling of families to reduce or prevent exposure to lead in the environment | DH21. Early identification of lead poisoning and counselling of families to reduce or prevent | PH23. Early identification of lead poisoning and counselling of families to reduce or prevent |

| | | | | | | |
|---|---|---|---|---|---|---|
| C16. Treatment of acute pharyngitis in children to prevent rheumatic fever | MHT16. Treatment of acute pharyngitis in children to prevent rheumatic fever | S19. Treatment of acute pharyngitis in children to prevent rheumatic fever | B20. Treatment of acute pharyngitis in children to prevent rheumatic fever | CHC21. Treatment of acute pharyngitis in children to prevent rheumatic fever | DH22.Treatment of acute pharyngitis in children to prevent rheumatic fever | PH24.Treatment of acute pharyngitis in children to prevent rheumatic fever |
| *C. Infectious Diseases Interventions = 18 of which 11 are MoPH high priority for implementation* | | | | | | |
| | | S20. Targeted age based and risk-based vaccinations for adults including tetanus, pneumococcus, influenza, hepatitis B, and any other relevant vaccinations | B21. Targeted age based and risk-based vaccinations for adults including tetanus, pneumococcus, influenza, hepatitis B, and any other relevant vaccinations | CHC22. Targeted age based and risk-based vaccinations for adults (including tetanus, pneumococcus, influenza, hepatitis B, and any other relevant vaccinations | DH23. Targeted age based and risk-based vaccinations for adults including tetanus, pneumococcus, influenza, hepatitis B, and any other relevant vaccinations | PH25. Targeted age based and risk-based vaccinations for adults including tetanus, pneumococcus, influenza, hepatitis B, and any other relevant vaccinations |
| ^C17. Mass helminthiases medicine administration | ^MHT18. Mass helminthiases medicine administration | ^S21. Mass helminthiases medicine administration | | | | |
| | | ^S22. Early detection and treatment of leishmaniasis | ^B22. Early detection and treatment of leishmaniasis | CHC23. Clinical diagnosis and treatment of Leishmaniasis with sodium stibogluconate (SSG) based on guideline | DH24. Clinical diagnosis and treatment of Leishmaniasis with sodium stibogluconate (SSG) based on guideline | PH26. Clinical diagnosis and treatment of Leishmaniasis with sodium stibogluconate (SSG) based on guideline |
| | | ^S23. HIV education and counselling, and provision of condoms for high-risk individuals | ^B23. Provider initiated testing and counselling for HIV, sexually transmitted infections, and hepatitis, including for adolescents, with rapid treatment of sexually transmitted infections, and referral for the immediate starting of treatment for those testing positive for HIV | ^CHC24. Provider initiated testing and counselling for HIV, sexually transmitted infections, and hepatitis, including for adolescents, with immediate treatment of sexually transmitted infections, provision of PrEP where | ^DH25. Provider initiated testing and counselling for HIV, sexually transmitted infections, and hepatitis, including for adolescents, with immediate treatment of sexually transmitted infections, provision of PrEP where relevant, preventive therapies for children born to mothers | ^PH27. Provider initiated testing and counselling for HIV, sexually transmitted infections, and hepatitis, including for adolescents, with immediate treatment of sexually transmitted infections, provision of PrEP where relevant, preventive therapies for children born to mothers |

| | | | | | | |
|---|---|---|---|---|---|---|
| | | | | relevant, and starting and on-going monitoring of appropriate treatment for those testing positive for HIV | with HIV, and starting and on-going monitoring of appropriate treatment for those testing positive for | with HIV, and the starting and on-going monitoring of appropriate treatment for those testing positive for HIV |
| | | | | CHC25. Following a new diagnosis of HIV, initial and annual screening for latent tuberculosis infection, initiation of isoniazid preventive therapy among all with positive screen but no evidence of active tuberculosis | DH26. Following a new diagnosis of HIV, initial and annual screening for latent tuberculosis infection, initiation of isoniazid preventive therapy among all with positive screen but no evidence of active TB | PH28. Following a new diagnosis of HIV, initial and annual screening for latent tuberculosis infection, initiation of isoniazid preventive therapy among all with positive screen but no evidence of active tuberculosis |
| | | | | ^CHC26. Provider initiated diagnosis of tuberculosis using sputum smear, and initiation of first line and second line treatment per current WHO guidelines for drug susceptible tuberculosis; referral for confirmation, assessment of drug resistance, and treatment of drug resistant tuberculosis | ^DH27. Confirmation, further assessment of drug resistance, and treatment of drug resistant tuberculosis | ^PH29. Drug susceptibility testing for cases of treatment failure and tertiary referral as needed; enrolment of those with MDR-TB for treatment |
| | | | | CHC27. For PLHIV and children under 5 who are close contacts | DH28. For PLHIV and children under 5 who are close contacts of individuals | PH30. For PLHIV and children under 5 who are close contacts of individuals |

| | | | | | | |
|---|---|---|---|---|---|---|
| | | | | of individuals with active TB, perform symptom screening, chest x-ray, and preventive therapy | with active TB, perform symptom screening, chest x-ray, and preventive therapy | with active TB, perform symptom screening, chest x-ray, and preventive therapy |
| | MHT19. Referral for HIV testing for diagnosed TB cases | S24. Referral for HIV testing for diagnosed TB cases | B24. Referral for HIV testing for diagnosed TB cases | ^CHC28. Screening for HIV in all individuals with a diagnosis of active tuberculosis; if HIV present, initiation of ARV treatment and HIV care | ^DH29. Screening for HIV in all individuals with a diagnosis of active tuberculosis; if HIV present, initiation of ARV treatment and HIV care | ^PH31. Screening for HIV in all individuals with a diagnosis of active tuberculosis; if HIV present, initiation of ARV treatment and HIV care |
| ^C18. In high and low prevalence areas use of rapid diagnostic test for the P. vivax and P. Falciparum malaria with treatment with relevant anti-malarial medicines based on National Treatment Guideline (NTG) | ^MHT20. Treatment of malaria diagnosed by rapid diagnostic test with relevant oral/rectal anti-malarial medicines based on NTG | ^S25. In high prevalence areas diagnosed by rapid tests and treatment with relevant anti-malarial medicines based on NTG | ^B25. Treatment of malaria diagnosed by rapid diagnostic test with relevant oral/rectal anti-malarial medicines based on NTG | ^CHC29. Treatment of malaria diagnosed by microscopy with relevant oral anti-malarial medicines based on NTG | ^DH30. Management of non-complicated and severe malaria including parenteral artesunate and full course of ACT based on NTG | ^PH32. Management of non-complicated and severe malaria including parenteral artesunate and full course of ACT based on NTG |
| C19. Provision of insecticide-treated nets to households in Malaria high-risk areas through mass campaign | MHT21. Provision of insecticide-treated nets to households in Malaria high-risk areas through mass campaign | S26. Provision of insecticide-treated nets to children and pregnant women in high-risk areas through Ante Natal Care visits (ANC) | B26. Provision of insecticide-treated nets to children and pregnant women in high-risk areas through Ante Natal Care visits (ANC) | CHC30. Provision of insecticide-treated nets to children and pregnant women in high-risk areas through Ante Natal Care visits (ANC) | DH31. Provision of insecticide-treated nets to children and pregnant women in high-risk areas through Ante Natal Care visits (ANC) | PH33. Provision of insecticide-treated nets to children and pregnant women in high-risk areas through Ante Natal Care visits (ANC) |

| | | | | | | |
|---|---|---|---|---|---|---|
| C20. In the context of an emerging infectious outbreak, disseminate advice and guidance on how to recognise early symptoms and signs and when to seek medical attention | MHT22. Only at time of risk for outbreak, basic case-based syndromic surveillance and reporting with contact with bodily fluids precautions | S27. Only at time of risk for outbreak, basic case-based syndromic surveillance and reporting with contact precautions | B27. Only at time of risk for outbreak, basic case-based syndromic surveillance and reporting with contact precautions | CHC31. Only at time of risk for outbreak, basic case-based syndromic surveillance and reporting with contact precautions | DH32. Case-based syndromic surveillance in emergency rooms/units and reporting with basic communicable disease isolation | PH34. Case-based syndromic surveillance in emergency rooms or units and reporting with advanced communicable disease isolation |
| | | | | | DH33. Diagnosis and vaccination for rabies | PH35. Diagnosis and vaccination for rabies |
| C21. Health education and counselling on HIV, TB and Malaria | MHT23. Health education and counselling on HIV, TB and Malaria | S28. Health education and counselling on HIV, TB and Malaria | B28. Health education and counselling on HIV, TB and Malaria | CHC32. Health education and counselling on HIV, TB and Malaria | DH34. Health education and counselling on HIV, TB and Malaria | PH36. Health education and counselling on HIV, TB and Malaria |
| C22. Identification and referral of presumptive TB cases | MHT24. Identification, referral, and sample transportation of presumptive TB cases | S29. Identification, referral, and sample transportation of presumptive TB cases | B29. Identification, referral, and sample transportation of presumptive TB cases | CHC33. Identification and diagnosis of presumptive TB cases, including identification of drug resistant TB strains | DH35. Identification and diagnosis of presumptive TB cases, including identification of drug resistant TB strains | PH37. Identification and diagnosis of presumptive TB cases, including identification of drug-resistant TB strains |

| | | | | | | |
|---|---|---|---|---|---|---|
| C23. Treatment and follow up of diagnosed TB cases and provision of IPT to TB contacts | MHT25. Treatment and follow up of diagnosed TB cases and provision of IPT to TB contacts | S30. Treatment and follow up of diagnosed TB cases and provision of IPT to TB contacts | B30. Treatment and follow up of diagnosed TB cases and provision of IPT to TB contacts | CHC34. Treatment and follow up of diagnosed TB cases and provision of IPT to TB contacts | DH36. Treatment of diagnosed TB cases and provision of IPT to TB contacts | PH38. Treatment of diagnosed TB cases and provision of IPT to TB contacts |
| C24. Active contact tracing of all Drug susceptible and Drug Resistant TB cases through household visits | MHT26. Active contact tracing of all Drug susceptible and Drug Resistant TB cases through household visits | S31. Active contact tracing of all Drug susceptible and Drug Resistant TB cases through household visits | B31. Active contact tracing of all Drug susceptible and Drug Resistant TB cases through household visits | CHC35. Active contact tracing of all Drug susceptible and Drug Resistant TB cases through household visits | | |
| *D. Chronic Non-Communicable Disease Interventions = 7 of which 3 are MoPH high priority for implementation* | | | | | | |
| | | | ^B32. Screening for diabetes among at-risk adults, and continuation of prescribed treatment, including for control of glycaemia, blood pressure and lipids, and consistent foot care | ^CHC36. Screening and management of diabetes among at risk adults, including initiation of prescriptions for glycaemic control, and management of blood pressure and lipids | ^DH37. Screening and management of diabetes among at risk adults, including initiation of prescriptions for glycaemic control, and management of blood pressure and lipids | ^PH39. Screening and management of diabetes among at risk adults, including initiation of prescriptions for glycaemic control, and management of blood pressure and lipids |
| | MHT27. Blood pressure measurement in those aged 40 years and above | S32. Periodic screening for hypertension for all adults and continuation of prescribed treatment | B33. Periodic screening for hypertension for all adults and continuation of prescribed treatment | CHC37. Initiation of treatment among individuals with severe hypertension, evidence of associated end organ changes or other high-risk factors | DH38. Initiation of treatment among individuals with severe hypertension, evidence of associated end organ changes or other high-risk factors | PH40. Initiation of treatment among individuals with severe hypertension, evidence of associated end organ changes or other high-risk factors |
| | | | | ^CHC38. On-going management and monitoring of chronic | ^DH39. On-going management and monitoring of chronic | ^PH41. On-going management and monitoring of chronic |

| | | | | | | |
|---|---|---|---|---|---|---|
| | | | | cardiovascular disease with continuation of prescribed treatment to reduce risk of further events | cardiovascular disease with continuation of prescribed treatment to reduce risk of further events | cardiovascular disease with continuation of prescribed treatment to reduce risk of further events |
| | | | B34. Chronic management of asthma and chronic obstructive pulmonary disorder with low dose inhaled corticosteroids and long acting bronchodilators | CHC39. Chronic management of asthma and chronic obstructive pulmonary disorder with low dose inhaled corticosteroids and long acting bronchodilators. | DH40. Management of acute exacerbations of asthma and chronic obstructive pulmonary disorder<br><br>*See also emergency care section* | PH42. Management of acute exacerbations of asthma and chronic obstructive pulmonary disorder |
| | | | | ^CHC40. Secondary prophylaxis with penicillin for rheumatic fever or established rheumatic heart disease | ^DH41. Secondary prophylaxis with penicillin for rheumatic fever or established rheumatic heart disease | ^PH43. Secondary prophylaxis with penicillin for rheumatic fever or established rheumatic heart disease |
| | | | | CHC41. Screening for breast cancer in all chronic disease diagnosis | DH42. Screening for breast cancer in all chronic disease diagnosis | PH44. Screening for breast cancer in all chronic disease diagnosis |
| | | | | CHC42. Early detection by visual inspection of early-stage cervical cancer, with referral | DH43. Early detection by visual inspection and treatment by cryotherapy and colposcopy of early-stage cervical cancer | PH45. Early detection by visual inspection and treatment by cryotherapy and colposcopy of early stage cervical cancer. |
| | | | B35. Awareness and referral of suspected cancer patients to the regional Hospital Oncology Wards for diagnosis and treatment | CHC43. Awareness, screening, and referral for suspected cancer patients to the regional Hospital Oncology | DH44. Awareness, screening, and referral for suspected cancer patients to the regional Hospital Oncology Wards for diagnosis and treatment | PH46 Awareness, screening, and referral for suspected cancer patients to the regional Hospital Oncology Wards for diagnosis and treatment |

| | | | | | | |
|---|---|---|---|---|---|---|
| | | | | Wards for diagnosis and treatment | | |
| *E. Mental, Neurological, and Substance Use Disorders Intervention = 18 of which 7 are MoPH high priority for implementation* | | | | | | |
| C25. Awareness, detection and referral of Common and severe mental health disorders | MHT28. Awareness, Detection, Basic psychosocial counselling, pharmacological treatment and referral of Common and severe mental health disorders | | | | | |
| | | | B36. Detection of anxiety disorders for all age groups using validated interview-based tools and referral for initiation of pharmacological treatment, referral for psychosocial support. | ^CHC44. Detection and referral for initiation of pharmacological treatment of all mental disorders for all age groups and continuation of psychosocial counselling<br><br>*See emergency section for clinically unstable patients* | ^DH45. Detection of common and severe disorders for all age groups and continuation of psychosocial counselling or psychotherapy with timely referral for initiation of pharmacological treatment<br><br>*See emergency section for clinically unstable patients* | ^PH47. Initiation of pharmacological and psychosocial counselling or psychotherapy for all mental health conditions |
| | | | B37. Detection of substance use disorders for all age groups using validated screening tools, and referral to drug demand reduction programme for pharmacological treatment and referral for psychosocial counselling | CHC45. Detection of substance use disorders for all age groups using validated screening tools, and referral to drug demand reduction programme for pharmacological treatment and | DH46. Referral to drug demand reduction treatment facility programme for pharmacological treatment and referral for psychosocial counselling or psychotherapy<br><br>*See emergency section for clinically unstable or acute (e.g.,* | PH48. Referral to drug demand reduction treatment facility for pharmacological treatment and referral to mental health hospital for psychosocial counselling or psychotherapy<br><br>*See emergency section for clinically unstable or acute (e.g.,* |

| | | | | | | |
|---|---|---|---|---|---|---|
| | | | | psychosocial counselling | *overdose, drug-induced psychosis, suicide, self-harm, and violence)* | *overdose, drug-induced psychosis, suicide, self-harm, and violence)* |
| | | | B38. Detection and follow up of psychotic disorders using validated interview-based tools with timely referral for management | CHC46. Detection, basic counselling and follow up of psychotic disorders using validated interview-based tools with timely referral for management, and continuation of psychosocial counselling for psychotic disorders | ^DH47. Prescription of pharmacological and psychosocial counselling for psychotic disorders especially bi-polar and schizophrenia conditions *See emergency section for clinically unstable e.g., severe acute agitation, suicide, self-harm, violence* | ^PH49. Prescription of pharmacological and psychosocial counselling for psychotic disorders especially bi-polar and schizophrenia conditions |
| C26. Community education to limit exposure to violence, including gender-based violence and conflict, and referral for appropriate care | MHT29. Active detection of exposure to gender-based violence and referral for appropriate care | S33. Active detection of exposure to gender-based violence and referral for appropriate care | B39. Active detection of exposure to gender-based violence and referral for appropriate care | ^CHC47. Psychosocial counselling for those exposed to violence<br><br>*See also emergency care section for medical support)* | ^DH48. Advanced management for effects of exposure to violence<br><br>*See also treatment for anxiety, depression, and emergency care section for medical support* | ^PH50. Advanced management of effects of exposure to violence<br><br>*See also treatment for anxiety, depression, and emergency care section for medical support* |
| | | | | CHC48. Continuation of prescribed pharmacological medicines and psychosocial counselling for epilepsy<br><br>*See emergency section for clinically unstable (e.g., active seizures)* | ^DH49. Prescription and initiation of pharmacological and psychosocial interventions for epilepsy<br><br>*Also see emergency section for clinically unstable (e.g., active seizures)* | ^PH51. Prescription and initiation of pharmacological and psychosocial interventions for epilepsy<br><br>*Also see emergency section for clinically unstable (e.g., seizures)* |

| | | | | | | |
|---|---|---|---|---|---|---|
| | | | | CHC49. Initiation of self-managed treatment using migraine protocol in acute phase | DH50. Initiation of self-managed treatment using migraine protocol in acute phase | PH52. Initiation of self-managed treatment using migraine protocol in acute phase |
| | | | | | DH51. Psychosocial support for patients with cancer | PH53. Psychosocial support for patients with cancer |
| *F. Emergency Care Interventions = 28 of which 13 are MoPH high priority for implementation* | | | | | | |
| ^C27. *Pre-hospital care*: User activated dispatch of basic ambulance services from district level | ^MHT30. *Pre-hospital care*: User activated dispatch of basic ambulance services from district level | ^S34. *Pre-hospital care*: User activated dispatch of basic ambulance services from district level | ^B40. *Pre-hospital care*: User activated dispatch of basic ambulance services from district level | ^CHC50. *Pre-hospital care*: User activated dispatch of basic ambulance services from district level | DH52. *Pre-hospital care*: User activated dispatch of basic ambulance services from district level | PH54. *Pre-hospital care*: User activated dispatch of basic ambulance services at provincial level |
| ^C28. *Pre-hospital care:* WHO basic emergency care - Initial syndrome-based management at scene by pre-hospital providers for difficulty in breathing, shock, altered mental status, and poly trauma | ^MHT31. Pre-*hospital care:* WHO basic emergency care - Initial syndrome-based management at scene by pre-hospital providers for difficulty in breathing, shock, altered mental status, and poly trauma | ^S35. *Pre-hospital care:* WHO basic emergency care - Initial syndrome-based management at scene by pre-hospital providers for difficulty in breathing, shock, altered mental status, and poly trauma | ^B41. *Pre-hospital care:* WHO basic emergency care - Initial syndrome-based management at scene by pre-hospital providers for difficulty in breathing, shock, altered mental status, and poly trauma | ^CHC51. *Pre-hospital care:* WHO basic emergency care - Initial syndrome-based management at scene by pre-hospital providers for difficulty in breathing, shock, altered mental status, and poly trauma | ^DH53. *Pre-hospital care:* WHO basic emergency care - Initial syndrome-based management at scene by pre-hospital providers for difficulty in breathing, shock, altered mental status, and poly trauma | ^PH55. *Pre-hospital care:* WHO basic emergency care - Initial syndrome-based management at scene by pre-hospital providers for difficulty in breathing, shock, altered mental status, and poly trauma |
| ^C29. *Pre-hospital care:* Direct provider monitoring during | ^MHT32. *Pre-hospital care:* Direct provider monitoring | ^S36. *Pre-hospital care:* Direct provider monitoring during | ^B42. *Pre-hospital care:* Direct provider monitoring during | ^CHC52. *Pre-hospital care:* Direct provider | ^DH54. *Pre-hospital care:* Direct provider monitoring | ^PH56. *Pre-hospital care:* Direct provider monitoring |

| | | | | | | |
|---|---|---|---|---|---|---|
| transport to appropriate health facility and structured handover to hospital personnel | during transport to appropriate health facility and structured handover to hospital personnel | transport to appropriate health facility and structured handover to hospital personnel | transport to appropriate health facility and structured handover to hospital personnel | monitoring during transport to appropriate health facility and structured handover to hospital personnel | during transport to appropriate health facility and structured handover to hospital personnel | during transport to appropriate health facility and structured handover to hospital personnel |
| | ^MHT33. Recognition of danger signs in neonates, children and adults, including early recognition of signs of serious infection and referral | ^S37. Recognition of danger signs in neonates, children and adults, including early recognition of signs of serious infection and referral | ^B43. Recognition of danger signs in neonates, children and adults, including early recognition of signs of serious infection and referral | ^CHC53. Recognition of danger signs in neonates, children and adults, including early recognition of signs of serious infection and referral | DH55. Triage of children and adults on arrival at facility with validated instrument (e.g. WHO/ICRC triage tool) | PH57. Triage of children and adults on arrival at facility with validated instrument (e.g. WHO/ICRC triage tool) |
| | | | | | DH56. Implementation of checklists for management of critically ill and injured patients in designated resuscitation area (WHO emergency and trauma care checklists) | PH58. Implementation of checklists for management of critically ill and injured patients in designated resuscitation area (WHO emergency and trauma care checklists) |
| ^C30. First aid: Interventions include airway positioning, choking interventions, and basic external haemorrhage control (direct pressure, tourniquet) | ^MHT34. First aid: Interventions include airway positioning, choking interventions, and basic external haemorrhage control (direct pressure, tourniquet), stabilization and referral | ^S38. Basic life support, plus protocol-based administration of oral fluids with adjustment for age and condition including malnutrition, stabilization and referral | ^B44. Basic syndrome-based management of difficulty breathing, shock, altered mental status, and poly trauma in dedicated emergency unit for neonates, children and adults {interventions include manual airway manoeuvres, oral/nasal airway placement, oxygen administration, bag-valve mask ventilation, temperature management, emergency administration of essential medications, including antibiotics for | ^CHC44. Basic syndrome-based management of difficulty breathing, shock, altered mental status, and poly trauma in emergency unit for neonates, children and adults {interventions include manual airway manoeuvres, oral/nasal airway placement, oxygen administration, bag-valve mask ventilation, | ^DH57. Advanced syndrome-based management of difficulty breathing, shock, altered mental status, and poly trauma in emergency unit, including for neonates, children and adults. (Interventions include intubation, mechanical ventilation, surgical airway, and placement of chest drain, haemorrhage control, defibrillation, administration of | ^PH59. Advanced syndrome-based management of difficulty breathing, shock, altered mental status, and poly trauma in emergency unit, including for neonates, children and adults. (Interventions include intubation, mechanical ventilation, surgical airway, and placement of chest drain, haemorrhage control, defibrillation, administration of |

| | | | | | | |
|---|---|---|---|---|---|---|
| | | | serious infection, stabilization and referral | temperature management, emergency administration of essential medications, including empiric antibiotics for serious infection, stabilization and referral | intravenous fluids via peripheral and central venous line with adjustment for age and condition, including malnutrition; emergency administration of essential medicines) | intravenous fluids via peripheral and central venous line with adjustment for age and condition, including malnutrition; emergency administration of essential medicines |
| | | | | CHC45. Management of severe acute exacerbations of asthma and chronic obstructive pulmonary disease {using systemic steroids, inhaled beta-agonists, and, if indicated, oral antibiotic and oxygen therapy | DH58. Management of acute ventilatory failure due to acute exacerbations of asthma and chronic obstructive pulmonary disease with use of bilevel positive airway pressure preferable | PH60. Management of acute ventilatory failure due to acute exacerbations of asthma and chronic obstructive pulmonary disease; in chronic obstructive pulmonary disease use of bilevel positive airway pressure preferable |
| | | | B45. Basic management of cardiovascular emergencies, including provision of aspirin for suspected acute myocardial infarction and external defibrillation after initial survey/assessment | CHC56. Basic management of cardiovascular emergencies, including provision of aspirin for suspected acute myocardial infarction and external defibrillation after initial survey/assessment | ^DH59. Advanced management of cardiovascular emergencies, including myocardial infarction, heart failure, acute arrhythmia, tamponade, and acute critical limb ischemia. {Interventions include aspirin, unfractionated heparin and thrombolytics, pacing and synchronized cardioversion, pericardiocentesis} | ^PH61. Advanced management of cardiovascular emergencies, including myocardial infarction, heart failure, acute arrhythmia, tamponade, and acute critical limb ischemia. {Interventions include aspirin, unfractionated heparin and thrombolytics, pacing and synchronized cardioversion, pericardiocentesis |

| | | | | | | |
|---|---|---|---|---|---|---|
| | | | | | | |
| | | | B46. Recognition of, and referral for, clinical hypoglycaemia | CHC57. Recognition and initial management of hypoglycaemia and hyperglycaemia | DH60. Recognition and management of hypoglycaemia and hyperglycaemia, including treatment of diabetic ketoacidosis | PH62. Recognition and management of hypoglycaemia and hyperglycaemia, including treatment of diabetic ketoacidosis |
| | | | | CHC58. Recognition of symptoms due e.g. to opioids/narcotics, sedative hypnotics or hallucinogens, including poisoning, acute intoxication and withdrawal symptoms, with referral for management | DH61. Recognition and management of symptoms due e.g. to opioids/narcotics, sedative hypnotics or hallucinogens, including poisoning, acute intoxication and withdrawal symptoms, | PH63. Recognition and management of symptoms due e.g. to opioids/narcotics, sedative hypnotics or hallucinogens, including poisoning, acute intoxication and withdrawal symptoms, |
| ^C31. Initial wound care, including bleeding control, cleaning and dressing | ^MHT35. Basic wound care, including bleeding control and suturing of simple lacerations | ^S39. Basic wound care, including bleeding control and suturing of simple lacerations | ^B47. Basic wound care, including bleeding control and suturing of simple lacerations | ^CHC59. Advanced wound care, including suturing of complex lacerations | ^DH62. Advanced wound care, including suturing of complex lacerations | ^PH64. Advanced wound care, including suturing of complex lacerations |
| | | | | ^CHC60. Minor soft tissue surgical procedure (drainage of simple abscess and removal of foreign body) | ^DH63. Minor soft tissue surgical procedure (drainage of simple abscess and removal of foreign body) | ^PH65. Minor soft tissue surgical procedure (drainage of simple abscess and removal of foreign body |
| ^C32. Splinting of acute fractures and dislocations | ^MHT36. Splinting of acute fractures and dislocations | ^S40. Splinting of acute fractures and dislocations | ^B48. Splinting of acute fractures and dislocations | ^CHC61. Reduction and non-operative management of acute fractures and dislocations | ^DH64. Reduction and non-operative management of acute fractures and dislocations, including traction | ^PH66. Reduction and non-operative management of acute fractures and dislocations, including traction |

| | | | | | | |
|---|---|---|---|---|---|---|
| | | | | | DH65. Management of ENT emergencies, including foreign body removal, peritonsillar abscess and epistaxis | PH67. Management of ENT emergencies, including foreign body removal, peritonsillar abscess and epistaxis |
| | | | | | DH66. Management of acute trauma of the eye, including acid and alkali burns | PH68. Management of acute trauma of the eye, including acid and alkali burns |
| *See also community based first aid and pre-hospital care* | | | | CHC62. Rapid scale up of service delivery capacity through provincial coordination | DH67. Protocol based mass casualty management and rapid scale up of service delivery capacity | PH69. Advanced protocol response based on provincial coordination for mass casualty management and rapid scale of service delivery capacity |
| *G. Surgical Interventions (not including obstetric surgery-- see maternal health) = 17 of which 11 are MoPH high priority for implementation* | | | | | | |
| | | | | | ^DH68. Burr hole to relieve acute elevated intracranial pressure | ^PH70. Burr hole to relieve acute elevated intracranial pressure |
| | | | | CHC63. Debridement and other treatment of soft tissue infection of small wounds | ^DH69. Debridement and other treatment of soft tissue infection (including diabetic foot) and osteomyelitis | ^PH71. Debridement and other treatment of soft tissue infection (including diabetic foot) and osteomyelitis |
| | | | | | DH70. Escharotomy or fasciotomy | PH72. Escharotomy or fasciotomy |
| | | | | | ^DH71. Trauma related amputations | ^PH73. Trauma related amputations |
| | *See emergency care section above* | | | | ^DH72. Reduction of acute fractures and dislocations, placement of external fixator and use of traction for fractures | ^PH74. Urgent surgical management of orthopaedic injuries (for example, by open reduction and internal fixation) |

| | | | | | | |
|---|---|---|---|---|---|---|
| | | | | | ^DH73. Irrigation and debridement of open fractures | ^PH75. Irrigation and debridement of open fractures |
| | | | | | ^DH74. Management of septic arthritis | ^PH76. Management of septic arthritis |
| | | | | | DH75. Basic skin grafting and release of contractures, including for burns | PH77. Basic skin grafting and release of contractures, including for burns |
| | | | | | ^DH66DH76. Relief of urinary obstruction by catheterization or suprapubic cystostomy | ^PH78. Relief of urinary obstruction by catheterization or suprapubic cystostomy |
| | | | | | ^DH77. Abdominal surgery including hernia repair, management of acute abdomen, removal of gallbladder, appendectomy, colostomy, management of hydatic cyst | ^PH79. Abdominal surgery including hernia repair, management of acute abdomen, removal of gallbladder, appendectomy, colostomy, management of hydatic cyst |
| | | | | | ^DH78. Trauma laparotomy | ^PH80. Trauma laparotomy |
| | | | B49. Early recognition and referral for congenital anomalies | | DH79. Early recognition and referral for congenital anomalies | ^PH81. Management of cleft lip/palate, club foot |
| | | | | | DH80. Simple ocular procedures e.g. foreign body removal | PH82. Basic ocular surgery, including cataract removal |
| | | | | | DH81. Basic dental procedures (treatment of caries, extraction, drainage of simple dental abscess) | PH83. Comprehensive dental procedures (treatment of caries, extraction, drainage of simple dental abscess) |
| *H. Palliative Care Interventions = 3* | | | | | | |

| | | | | | | |
|---|---|---|---|---|---|---|
| | MHT37. Oral palliative care and pain control measures with non-opioid agents | | B50. Oral palliative care and pain control measures with non-opioid agents | CHC64. Oral and parenteral palliative care and pain control measures with non-opioid agents | DH82. Treatment of severe acute pain including in association with procedures, including with opioid and non-opioid agents | PH84. Treatment of severe acute pain with opioid and non-opioid agents |
| | | | | | DH83. Procedural sedation | PH85. Procedural sedation |
| *I. Rehabilitation Interventions = 2* | | | | | | |
| | MHT38. Identification of people with disabilities and referral to nearest services for physical rehabilitation or physiotherapy treatment in mobile vehicle | S41. Identification of people with disabilities and referral to nearest services for physical rehabilitation | B51. Identification of people with disabilities and referral to nearest services for physical rehabilitation | CHC65. Identification of people with disabilities and referral to nearest services for physical rehabilitation | DH84. Physical mobilization and strengthening activities following acute injury or illness and guidance in use of rehabilitation equipment (e.g., crutches, wheelchair etc.) | PH86. Physical mobilization activities and provision of appropriate rehabilitation equipment (e.g., crutches, wheelchair etc.) |
| Sub-total number of interventions at the 7 levels of the health system outlined in this table 1 = 331 | | | | | | |
| 25 interventions at community level:<br>9 = HPI ***<br>9 = EUHC****<br>7 = country context specific | 27 interventions by mobile health team:<br>15 = HPI<br>5 = EUHC<br>7 = country context specific | 34 interventions at sub-health centre level:<br>16 = HPI<br>8 = EUHC<br>10 = country context specific | 42 interventions at basic health centre level:<br>17 = HPI<br>10 = EUHC<br>15 = country context specific | 54 interventions at comprehensive health centre level:<br>27 = HPI<br>17 = EUHC<br>10 = country context specific | 74 interventions at district hospital level:<br>41 = HPI<br>23 = EUHC<br>10 = country context specific | 75 interventions at provincial hospital level:<br>41 = HPI<br>25 = EUHC<br>9 = country context specific |
| Some of the sub-total of all 331 interventions at the 7 levels of the health system in the above table 1 are repeated at different levels of the health system e.g. see number S1 at the sub-health centre which is also an intervention (B1) at the basic health centre level in section A, the reproductive, maternal and newborn health section. So a total of the different types of interventions is given below. | | | | | | |

| |
|---|
| TOTAL NUMBER OF DIFFERENT INTERVENTIONS = 149 (138 in table 1 + 11 population-based interventions in table 2) many of which were previously in the BPHS and the EPHS. The interventions reflect the epidemiological profile in the country and the fact that there are still too many deaths among mothers and the newborn. Of the 138 different types of interventions in table 1, 92 constitute essential universal health care interventions (EUHC as defined in DCP3), of which 81 are high priority for implementation. The 92 EUHC interventions are based on international and/or local evidence of effectiveness, cost-efficiency, and feasibility of implementation. If implemented effectively there should be an improvement in equitable access and significant outcomes; they will also contribute to adding quality to health services. The remaining 43 interventions are country context specific. Plus 15 inter-sectoral inter-ministerial policy interventions to reduce behavioural and environmental risks for early design and implementation - see table 3. The costs of the inter-sectoral inter-ministerial policy interventions are not included in the cost of the IPEHS. Only the interventions in tables 1 and 2 have been costed. |

* As of BPHS and EPHS staffing – the list of staff will be reviewed by the MoPH to determine if there are sufficient types and numbers of staff to implement the IPEHS. Once it is decided if, the ministry may need to have discussions with the Civil Service Commission as there may be cost and formal recognition of some disciplines of staff

** The letter in capital letters at the beginning of an intervention refers to the level of the health system or type of health facility e.g. C = community, DH = district hospital etc.

^**BOLD.** Where and/or when there is on-going armed conflict or resources are low those interventions or a component of an intervention in **bold** are **high priority interventions (HPI)** for the Ministry of Public Health – listed as highest priority platform (HPP) in tables 1-4 in DCP3 Annex 3C, 2017, Essential Universal Health Coverage: Interventions and Platforms in Disease Control Priorities. DCP3. World Bank, Washington. http:dcp-3org/ 2017, Essential Universal Health Coverage: Interventions and Platforms in Disease Control Priorities. DCP3. World Bank, Washington. http:dcp-3org/

****EUHC = Essential universal health care in Disease Control Priorities (DCP3), World Bank, Washington. http:dcp-3org/

**Ministry of Public Health, Afghanistan**

**Integrated Package of Essential Health Services 2021:**

# Population-based Interventions

**Table 2**

**P1. Mass media messages, especially radio and television, concerning healthy eating, physical activity, and mental well being****
**P2. Systematic identification of individuals with TB symptoms among high risk groups**
P3. Mass media messages concerning use of tobacco, alcohol, and other addictive products
P4. Mass media messages, concerning awareness about hand washing and health effects of household/indoor air pollution
P5. Conduct simulation exercises with, and awareness raising among, health personnel for disease outbreaks including outbreak investigation, contact tracing, and emergency response
P6. Ensure plan in place to ensure ability to cope with large increase of patients due to infectious diseases e.g. stockpiles of disinfectants, equipment for patient care, and personal protective equipment
P7. Ensure influenza vaccine available at all levels of the health system
P8. In high malaria transmission settings, targeted vector control strategies
P9. Develop plans and legal standards for reducing interactions between infected persons and uninfected population, and implement and evaluate infection control measures
P10. Conduct simulation exercises for response to armed conflict emergencies
P11. Ensure preparedness strategy to have all in place for surge capacity in hospital beds, stockpiles of disinfectants, equipment for supportive care and personal protective equipment

**The two population based interventions written i**n bold** are those that where and/or when there is on-going armed conflict and/or resources are low are highest priority interventions (HPI) for the Ministry of Public Health – listed as highest priority platforms (HPP) in annex 3C 1-4 in DCP3 Annex 3C, 2017, Essential Universal Health Coverage: Interventions and Platforms in Disease Control Priorities. DCP3. World Bank, Washington. http:dcp-3org/
Population-based interventions numbers 1-9 are also in Table 3 annex 3C, DCP3 2017. Numbers 10 and 11 are country specific because of the extent of emergencies and trauma in the country

**Ministry of Public Health, Afghanistan**

**Integrated Package of Essential Health Services 2021:**

# Top Priority Inter-Sectoral, Inter-Ministerial Policy Interventions

**Table 3**

In this table 3 is a list of 15 highest priority urgent inter-sectoral policy interventions for early implementation for the prevention of ill health and to reduce health related poverty. They also address the SDGs. The interventions were decided at inter-ministerial meetings in Kabul during 2018 using DCP3 volume 9 in which table 2.3 has a total of 29 early policy interventions. At the time of the publication of this document policy intervention number 1 below is the highest priority to be addressed based on the high levels of air pollution in the country especially in cities. The remaining policy interventions are not in order of priority and ideally all need to be addressed concurrently.

The policy interventions have the strongest international evidence and the highest likely magnitude of health effect in the country. In some countries the policies have quickly and directly resulted in a measurable decline in mortality. The interventions are also likely to provide best value for money and be feasible in the low income context of Afghanistan.

**Top priority inter-sectoral, inter-ministerial policy interventions***

1. ***Air pollution*: regulate transport, industrial, power, and household generation emissions**
2. *Public transportation*: build and strengthen affordable public transport systems in urban areas
3. *Substance use:* impose high taxes on tobacco, cigarettes and other addictive substances
4. *Substance use:* impose strict regulation of advertising, promotion, packaging, and availability of tobacco, cigarettes and other addictive substances, with enforcement
5. *Smoking in public places:* ban smoking in public places
6. *Food quality:* ensure that foods have adequate nutritional value
7. *Iron and folic acid:* fortify food
8. *Iodine:* Fortify food
9. *Trans fats:* ban and replace with polyunsaturated fats
10. *Salt:* impose regulations to reduce salt in manufactured food products
11. *Sugar sweetened drinks:* tax to discourage use
12. *Salt and sugar*: provide consumer education against excess use, including product labelling
13. *Vehicle safety:* enact legislation and enforcement of personal safety measures, including seat belts in vehicles and helmets for motorcycle and bicycle users
14. *Pesticides:* enact strict control and ban highly hazardous pesticides
15. *Water and sanitation:* enact standards for safe drinking water, sanitation and hygiene within households, institutions and business companies
